# Subclinical Enthesopathy in Psoriasis—An Ultrasonographic Study

**DOI:** 10.3390/medsci12030040

**Published:** 2024-08-16

**Authors:** Rucsandra Cristina Dascălu, Andreea Lili Bărbulescu, Ștefan Cristian Dinescu, Cristina Elena Biță, Loredana Elena Stoica, Florentin Ananu Vreju

**Affiliations:** 1 Department of Rheumatology, Faculty of Medicine, University of Medicine and Pharmacy of Craiova, 200349 Craiova, Romania; rucsandrag@gmail.com (R.C.D.); stefan.dinescu@umfcv.ro (Ș.C.D.); cristina.gofita@umfcv.ro (C.E.B.); florentin.vreju@umfcv.ro (F.A.V.); 2 Department of Pharmacology, Faculty of Medicine, University of Medicine and Pharmacy of Craiova, 200349 Craiova, Romania; 3 Department of Dermatology, Faculty of Medicine, University of Medicine and Pharmacy of Craiova, 200349 Craiova, Romania; loredana.stoica@umfcv.ro

**Keywords:** psoriasis, ultrasonography, enthesis, MASEI

## Abstract

The present study is aimed at assessing the presence and prevalence of subclinical entheseal changes in Psoriasis (PsO) patients using musculoskeletal ultrasonography (US), conjoined with the analysis of possible differences in terms of demographic, clinical, or biological features. We carried out an observational study on 54 patients with PsO and 40 controls. Subclinical enthesopathy, according to OMERACT definitions, was identified in 20 of the psoriasis patients (37.03%), a significantly difference compared to the controls (5 patients; 10.20%). A comparison between US examinations for psoriasis patients and controls indicates that all the examined areas manifested changes in a significantly higher percentage of patients than the controls. The most common structural changes were represented by thickened tendon (85%), calcification (65%), erosions (35%), power Doppler (PD) signal (20%), and bursitis (5%). The difference in mean MASEI (Madrid Sonographic Enthesitis Index) score between the psoriasis and control groups was statistically significant (10.56 + 2.96 vs. 2.9 + 2.20; *p* < 0.0001). In conclusion, ultrasound is an easily accessible and vital follow-up method for psoriasis patients to enable an early, subclinical detection of entheseal involvement, i.e., the first red-flag sign for a future transition to psoriatic arthritis (PsA).

## 1. Introduction

Psoriasis (PsO), one of the few common and specific-enough skin diseases to be identified in clinical practice, exhibits various articular symptoms in the absence of sufficient additional criteria to establish diagnosis in up to 30% of the cases of psoriatic arthritis (PsA) [1,2,3,4]. Enthesitis, the hallmark of PsA, is difficult to detect during clinical examination as it can vary from asymptomatic to inflammatory, mechanical, or traumatic manifestations [5,6]. The factors involved in the complex progression towards inflammatory arthritis are not entirely clear. There is a specific input of genetic, epigenetic, and immunological contributions, aligned with environmental and demographic ones, with a complex orchestration of molecular and cellular mechanisms [7,8,9]. In the subclinical phase, the lack of symptoms determines undercover progressive damage and the development of PsA, driven by the multifaceted interplay of inflammatory pathways with destructive, irreversible damage [6]. Although remarkable advances have enabled the development of targeted therapies and delayed progression to inflammatory arthritis, intercepting and defining the onset of PsA remains challenging, and prospective research remains mandatory [10,11].

Regarding subclinical detection, specialists would face quite an enigma during clinical examination without imaging techniques, such as ultrasonography (US), which are used to detect the type and nature of the changes properly. US has been developed as a top-level tool to evaluate all kinds of rheumatic pathologies, enjoying the advantage of being non-invasive, reproducible, and easily used by experienced examiners [12,13,14,15]. Thus, this technique elevates clinical examination, conventional radiography, and even M.R.I., especially during the early stages of enthesopathic changes [16]. To evaluate the extent of entheseal abnormalities, several scores, including the MASEI score, which includes power Doppler and upper limb examination, have been tested and validated as applicable procedures in detecting signs of both subclinical and constituted disease [17,18].

To develop our study, we set out to assess the presence and prevalence of subclinical entheseal changes in psoriasis patients via musculoskeletal ultrasonography (US) to detect the cases at risk of PsA development, concurrently with the analysis of possible differences in terms of demographic, clinical, or biological features of the disease. Identifying cases at risk for this transition provides a better picture of patients’ clinical assessment and enables clinicians to apply a personalized therapeutic approach to prevent or delay the development of PsA.

## 2. Materials and Methods

We conducted an observational study on 54 patients with PsO admitted into the Dermatology Unit of the Craiova Emergency County Hospital, Romania, between August 2023 and January 2024. The selection criteria focused on subjects over 18 years old, already recorded in the Dermatology Unit with a psoriasis diagnosis, and the administration of systemic therapy (synthetic or biologic drugs). Patients with clinical signs of arthritis and/or enthesitis, surgical interventions, or local injections to the examined entheseal areas, trauma, or a history of inflammatory rheumatic conditions were excluded from the study. Following the study protocol, we collected data that included demographic, clinical, and laboratory parameters, and imagistic methods. The control group included 40 patients with similar demographic characteristics and no history of autoimmune conditions.

All participants signed an informed consent form before their inclusion in the study. The study was performed under the ethical principles of the Helsinki Human Right’s Declaration and was approved by the Ethics Committee of the Craiova Emergency County Hospital (registration no. 216/21.09.2023).

We assessed the severity of psoriatic skin lesions related to the Psoriasis Area and Severity Index (PASI) [19], where scores are multiplied by the disease severity score and the weighting for each body area, yielding a score between 0 and 72: mild (score < 7), moderate (score 7–15), and severe psoriasis (score > 15).

The US examination was performed by an expert sonographer, blinded to the history, clinical findings, and biology of each patient. We resorted to an Esaote MyLab 25 machine equipped with a high-frequency linear probe (10–18 MHz). Each enthesis was scanned bilaterally in longitudinal and axial planes, in grayscale, to detect morpho-structural changes and subsequently with the power Doppler (PD) technique to detect abnormal blood flow, as defined by the Outcome Measures in Rheumatology (OMERACT) Ultrasound Task Force [13]. The MASEI score was used to quantify the extent of ultrasonographic enthesis abnormalities [20]. The examined areas envisaged sections such as the triceps tendon, quadriceps tendon, proximal and distal patellar tendon, Achilles tendon, and plantar fascia. For each enthesis, we assessed the presence or absence of the following changes: thickening, calcifications/enthesophytes, erosions, and the PD signal. The presence of retrocalcaneal bursitis was evaluated as a component of the MASEI index.

We developed the statistical analysis via GraphPad Prism 5.5. The results were presented as mean ± S.D., following our data analysis based on the *t*-test and the one-way ANOVA to compare the groups and Pearson/Spearman’s coefficient to evaluate correlations. We considered a level of *p* < 0.05 as statistically significant.

## 3. Results

Our analysis included 54 consecutive PsO patients and 40 controls. The PsO group consisted of 26 female (48.14%) and 28 male (51.86%) patients, similar to the distribution of controls (47.5% female and 52.5% male). The mean age of patients with PsO was 43.4 years and 41.86 years for the control group. The average disease duration was 5.1 years.

The general characteristics of the studied groups are presented in Table 1.

**Figure 1:**
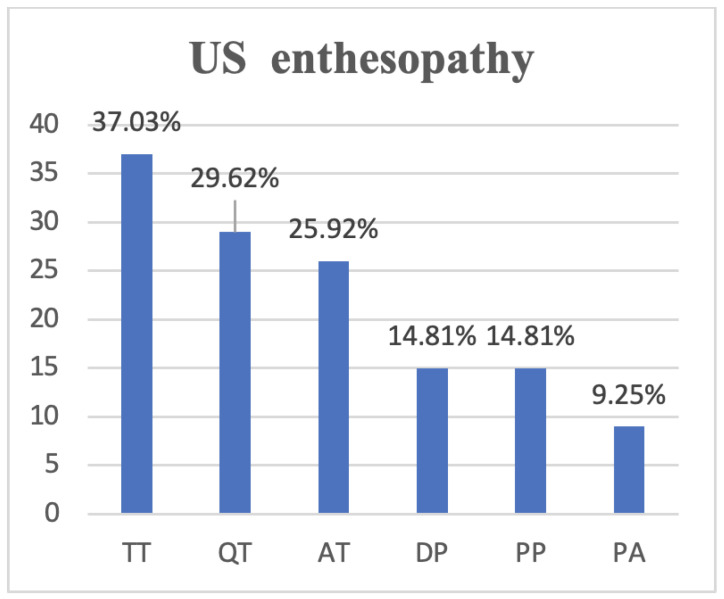
Enthesitis distribution in the PsO study group. TT, triceps tendon; QT, quadriceps tendon; PP, proximal patellar tendon; DP, distal patellar tendon; AT, Achilles tendon; PA, plantar aponeurosis.

Subclinical enthesopathy, according to OMERACT definitions, was identified in 20 of the psoriasis patients (37.03%), a significant difference compared to controls (5 patients; 10.20%) (Figure 1). The most common site was represented by the triceps tendon (TT) (20; 37.03%), followed by the quadriceps tendon (QT) (16; 29.62%%), Achilles tendon (AT) (14; 25.92%), proximal patellar tendon (PP) (8; 14.81%), distal patellar tendon (DP) (8; 14.81%), and plantar aponeurosis (PA) (5; 9.25%) (Figure 1).

**Figure 2:**
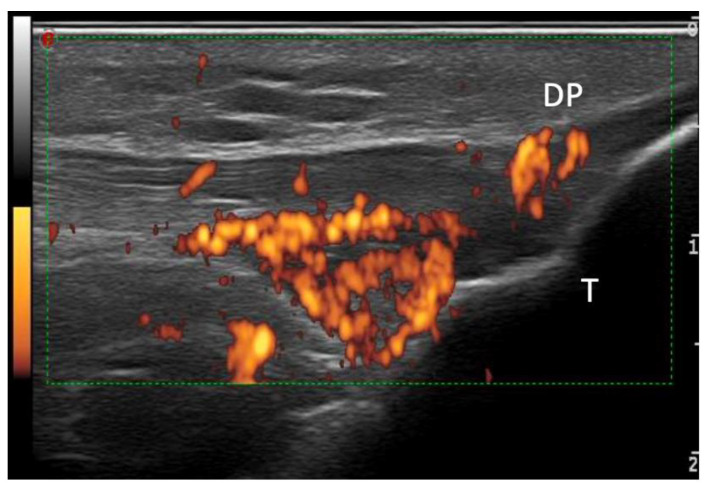
Power Doppler US image of the patellar tendon’s distal enthesis, showing a hypoechoic tendon, with the loss of a homogenous fibrillar pattern primarily near the cortical bone (<2 mm), with an intense power Doppler signal. DP—distal patellar tendon; T—tibial tuberosity.

The most common structural changes were represented by the thickened tendon (85%), calcification (65%), erosions (35%), PD (20%) (Figure 2), and bursitis (5%).

**Figure 3:**
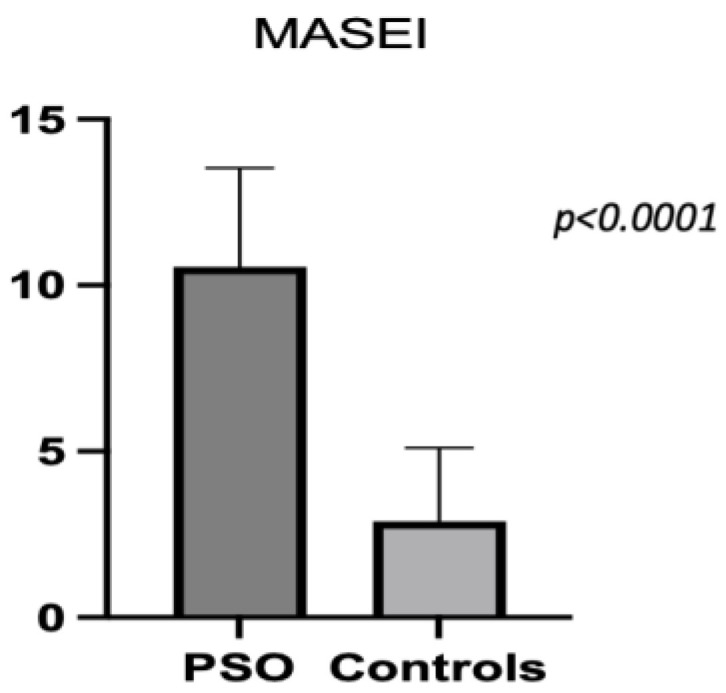
Mean MASEI score in PsO and controls.

The difference in the mean MASEI score between the psoriasis and control groups was statistically significant (10.56 ± 2.96 vs. 2.9 ± 2.20; *p* < 0.0001) (Figure 3).

A comparison between US examinations for psoriasis patients and controls revealed that all the examined areas manifested changes in a significantly higher percentage than the controls (Table 2). Also, regarding the identified structural abnormalities, we recorded that for increased tendon thickness, the presence of calcifications and erosions showed higher rates in psoriasis patients when compared to the control group. No significant difference was obtained for PD signal or bursitis (Table 2).

Table 3 contains an overview of the distribution of US changes depending on the involved situs.

**Figure 4:**
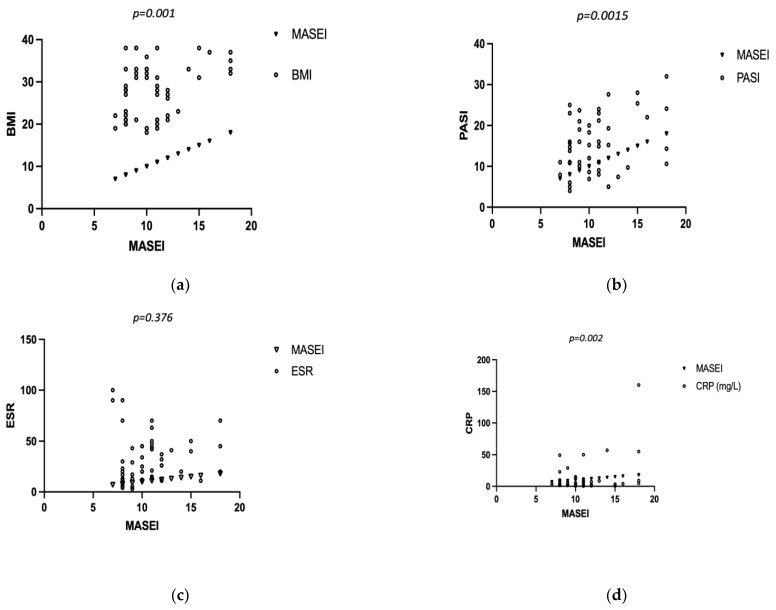

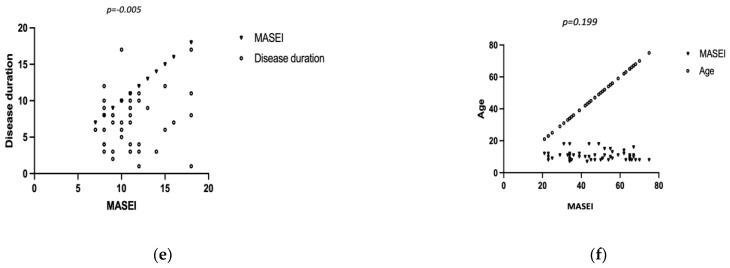
MASEI score and (**a**) BMI; (**b**) PASI; (**c**) ESR.; (**d**) CRP; (**e**) age; and (**f**) disease duration.

Further statistical analysis was conducted to analyze the possible correlation between certain disease-associated variables, demographic factors, and the MASEI score. No association was revealed for disease duration (r = −0.005, *p* = 0.702), age (r = 0.199, *p* = 0.147), or ESR (r = 0.12, *p* = 0.376). We obtained a moderate correlation for BMI (r = 0.41, *p* = 0.001), CRP (r = 0.406, *p* = 0.002), and PASI (r = 0.32, *p* = 0.015) (Figure 4a–e).

## 4. Discussion

Entheseal involvement constitutes the primary pathological process of psoriatic arthritis, and it has been argued that up to one-third of psoriasis patients develop this association with disease evolution [1,8,9]. Chronic continuous inflammatory processes in psoriatic derma, joints, and enthesis display common pathologic cellular and vascular immune abnormalities [20,21]. The early detection of entheseal changes, using the US Doppler technique, is of the utmost importance for an early accurate diagnosis and detection of the transition of psoriasis patients towards PsA.

The current study analyzed the use of entheseal US for detecting subclinical changes and the variables that might affect the presence of enthesopathy in PsO patients. Several reports have proven that ultrasonography, an easily accessible and reproducible method, ranks as an essential technique in assessing entheseal structural changes even in subclinical stages, with a subsequent early diagnosis and individualized follow-up.

The reported prevalence of asymptomatic US enthesopathy varies between published research [22,23,24,25,26,27]. Our study detected the presence of subclinical enthesopathy in 37.03% of cases, similar to the reports of Moshrif et al. [22], De Filips et al. [23], and Eder et al. [24]. Higher prevalence was noticed by Vyas et al. in 2020, with a percentage of 62% [25], while Acquacalda found that 46% of the 34 included patients displayed structural entheseal changes during US examination [26]. However, a lower prevalence was noted in the study by Naredo et al., 11.6% [27].

Yet, the issue of the most common site of early detection of subclinical enthesopathy in psoriasis patients remains controversial. Although the subject-specific literature mentions the involvement of lower limb extremities, a widespread variability between studies has been reported [24,25,28,29,30,31]. The most common site following our study was the triceps tendon (37.03%), followed by the quadriceps tendon (29.62%), Achilles tendon (25.92%), the proximal patellar tendon, and the distal patellar tendon with similar prevalence (14.81%), and plantar aponeurosis (9.25%). The incidence of enthesopathy was significantly higher in the triceps tendon. Although several studies focus on the US evaluation of lower extremities, the thickness of TT is a valuable indicator as it can be involved in the early stages of the disease. Hussein et al. found the distal insertion of the patellar tendon as the most frequent site involved (68.8%) with at least one pathological US finding, followed by proximal patellar tendon insertion (67.5%), quadriceps tendon insertion (53.1%), plantar fascia insertion (39.4%), and Achilles tendon insertion (34.4%) [28]. In the same line of thought, Eder et al. [24] and Di Matteo et al. [29] report the same phenomenon. However, a different distribution of enthesitis was reported by Moshrif et al. [22], Gutierrez et al. [30], and Vyas et al. [25], as researchers here found that Achilles enthesis is the most common site for enthesitis via US. Zuliani et al. reported that the distal patellar tendon is the most frequent site of enthesitis. They concluded that the most common abnormality is represented by an increased thickness [31]. It is difficult to establish the most common site of enthesis involvement based on academic reports, given the high degree of variability in terms of population features and the degree of wear and tear of the upper and/or lower limb(s) during daily activities and occupational practices. However, the US evaluation must cover all the areas of interest for a proper view of entheseal involvement.

After analyzing several structural tendon changes, we found a significantly different prevalence of chronic changes, such as calcifications and erosions, between psoriasis patients and controls. An increased tendon thickness was the most commonly reported finding in our study group, followed by calcifications, erosions, PD enthesitis, and bursitis, with the lowest prevalence. Graceffa et al. agree that the same prevalent abnormality exists [32]. Acquitter et al. reported the most common structural change was the presence of enthesophytes in 13.3% of the population [33]. Gisondi et al. published similar reports, arguing that these changes were more frequent in psoriatic patients than controls [34]. Karamanlioglu et al. revealed that tendon thickness, erosion, and calcification were significantly higher in the psoriasis group than in the control group [35]. The study by Hussein et al. reported that entheseal thickening was the most frequent US abnormality [28]. McGonagle et al. in [36] describe a similar finding as secondary to the initial stage of tendon edema. Given that the report focused on lower limb entheseal analysis, it was suggested that the length, anatomy, and physiology of the entheses may each contribute to the differential distribution. Consequently, enthesitis, partly due to mechanical stress forces, may appear first at the distal entheseal sites of the lower limbs [37].

We recorded a significantly higher mean MASEI score in the PsO group compared to the controls. Likewise, Naredo et al. [27], Vyas et al. [25], and Karamanliogluet al. [35] mention a similar finding in their previously published reports. Further, we developed our statistical analysis to establish possible correlations between demographic and disease-associated variables and MASEI scores. A study on 490 psoriasis patients, published by Chen et al. in 2023, validated that age, BMI, and psoriasis severity represented independent risk factors related to enthesitis and synovitis. Additionally, they concluded that there was no interrelation between the severity of skin lesions and subclinical or clinical PsA stages [38]. Our study identified a moderate, positive correlation between the MASEI score and BMI and PASI score, the most widely used scale for assessing the severity of psoriasis. Also, we noted a correlation between the CRP and MASEI score. No notable relationship was recorded for age, disease duration, or ESR. Moshrif et al. identified a positive correlation between enthesitis and BMI and age [22]. Zuliani et al. [31] reported an association between enthesis inflammation and PASI score, suggesting the close interplay between skin and enthesis throughout disease evolution. In a similar vein to our findings, Gisondi et al. observed that enthesitis is directly linked to BMI, but not to age or disease duration [34]. Although previous reports, such as the one authored by Ash et al. [39], underlined the presence of augmented enthesopathy scores for patients with nail psoriasis as a mark of systemic subclinical entheseal abnormalities, our data did not reveal any association. Our findings are similar to those of several other previous studies.

Various field-related researchers have provided evidence that subclinical entheseal changes are present in patients with psoriasis, and our data confirm the presence of such changes related to subclinical enthesis inflammation and transition to psoriatic arthritis. Using ultrasound as an efficient and accessible method to assess structural changes is mandatory for our patients’ follow-up. The relatively low number of patients included in our study is a limitation, with an impact on the calculated correlations between certain demographic and disease-related variables and US changes, thus, a multicentric extension is advisable for further research. Also, another limitation of our study is the lack of subgroup analysis relative to the current/previous specific medication, which can impact the degree of systemic inflammation and, subsequently, the presence of enthesitis. Consequent to the relatively low number of patients, the differentiation based on the class and type of specific medication cannot be performed, as it cannot present statistical significance.

## 5. Conclusions

Ultrasound emerges as an undoubtedly accessible and important follow-up method in psoriasis patients, enabling the early subclinical detection of entheseal involvement, i.e., the first red-flag sign for a future transition to PsA. Subsequently, an early and precise diagnosis provides a more appropriate risk stratification and individualized therapeutic management.

## Figures and Tables

**Table 1:** General characteristics of the study groups.

	PsO (N = 54)	Controls (N = 40)	*p*
Sex Female (N; %) Male (N; %)	26 (48.14%) 28 (51.86%)	19 (47.5%) 21 (52.5%)	0.320
Age (years) (mean; SD)	43.4; 14.5	41.86; 8.5	0.323
Disease duration (years) (mean; SD)	5.6; 7.5	-	-
BMI (kg/m^2^sc) (mean; SD)	27.8; 3.23	26.9; 5.32	0.179
Smoking (N; %) Never Current Previous smoking	10 15 29	18 10 12	0.023
Diabetes	7	1	0.002
Hypertension	18	10	0.32
Metabolic syndrome	10	2	0.004
Psoriasis type (N; %) Nail Skin Nail and skin	6; 11.11% 54; 100% 6; 11.11%	-	
Therapy sDMARDs (N; %) bDMARDs/tsDMARDs (N; %)			
	34; 62.96%		
	20; 37.04%		
PASI	15.24 ± 6.92	-	-
MASEI score	10.56; 2.96	2.9; 2.1	<0.0001
E.S.R. (mm/h)	31.34 ± 23.29	11.21 ± 3.55	0.002
CRP (mg/L)	11.23 ± 24.75	3.23 ± 1.21	<0.001
SUA (mg/dL)	6.23 ± 2.23	3.45 ± 1.27	0.02

BMI—body mass index; cs/b/tsDMARDs—conventional synthetic/biologic/targeted synthetic disease-modifying antirheumatic drug; PASI—Psoriasis Area Severity Index; MASEI—Madrid Sonographic Enthesis Index; ESR—erythrocyte sedimentation rate; CRP—C-reactive protein.

**Table 2:** Comparison of US examination between study groups.

	PsO (N = 54)	Controls (N = 40)	*p*
MASEI score (mean; SD)	10.56 + 2.9	2.9 + 2.2	<0.0001
TT (mean; SD)	2.71 + 2.05	1.51 + 1.06	0.021
QT (mean; SD)	2.34 + 2.5	0.71 + 0.51	<0.001
PP (mean; SD)	1.71 + 2.02	0.91 + 0.99	<0.001
DP (mean; SD)	1.51 + 1.91	0.71 + 0.71	0.003
AT (mean; SD)	1.43 + 1.2	0.51 + 0.32	0.002
FP (mean; SD)	0.72 + 1.41	0.21 + 0.4	0.039
Structural abnormalities (mean; SD)	1.97 + 3.25	0.52 + 0.62	0.005
Thickened tendon (mean; SD)	3.65 + 2.1	1.87 + 1.21	<0.001
Enthesis calcification/ enthesophyte (mean; SD)	1.21 + 1.05	0.21 + 0.43	<0.001
Erosions (mean; SD)	0.51 + 0.21	0.1 + 0.21	0.001
Enthesis PD (mean; SD)	0.41 + 0.43	0.15 + 0.12	0.212
Retrocalcaneal bursitis (mean; SD)	0.04 + 0.32	0	0.32

**Table 3:** US changes according to MASEI.

	Thickened Tendon N = 17	Erosions N = 7	Enthesis Calcification/ Enthesophyte N = 13	Enthesis PD n (%) N = 4	Bursitis N = 1
TT (N; %)	5 (29.41%)	0 (0.0%)	3 (23.07%)	1 (25%)	0 (0.0%)
QT (N; %)	2 (11.76%)	0 (0.0%)	2 (15.38%)	1 (25%)	0 (0.0%)
PP (N; %)	2 (11.76%)	0 (0.0%)	1 (7.69%)	0 (0.0%)	0 (0.0%)
DP (N; %)	3 (17.64%)	0 (0.0%)	1 (7.69%)	0 (0.0%)	0 (0.0%)
PA (N; %)	1 (5.88%)	2 (28.57%)	0 (0.0%)	0 (0.0%)	0 (0.0%)
AT (N; %)	4 (23.52%)	5 (71.43%)	6 (46.15%)	2 (50%)	1 (100%)

MASEI, Madrid Sonography Enthesitis Index; TT, tibial tuberosity; QT, quadriceps tendon; PP, proximal patellar tendon; DP, distal patellar tendon; AT, Achilles tendon; PA, plantar aponeurosis. Enthesitis PD was defined as the presence of the power Doppler signal within the enthesis at less than 2 mm from the bone cortical. Bursitis was described as a hypoechoic/anechoic area at the level of a specific bursa or tendon (Esaote, MyLab25 18 MHz).

## Data Availability

The dataset is available on request from the authors.
